# Cellular Barriers after Extravasation: Leukocyte Interactions with Polarized Epithelia in the Inflamed Tissue

**DOI:** 10.1155/2016/7650260

**Published:** 2016-01-28

**Authors:** Natalia Reglero-Real, Diego García-Weber, Jaime Millán

**Affiliations:** ^1^Centro de Biología Molecular Severo Ochoa, CSIC-UAM, Campus Cantoblanco, 28049 Madrid, Spain; ^2^William Harvey Research Institute, Barts and The London School of Medicine and Dentistry, Queen Mary University of London, Charterhouse Square, London, EC1M 6BQ, UK

## Abstract

During the inflammatory response, immune cells egress from the circulation and follow a chemotactic and haptotactic gradient within the tissue, interacting with matrix components in the stroma and with parenchymal cells, which guide them towards the sites of inflammation. Polarized epithelial cells compartmentalize tissue cavities and are often exposed to inflammatory challenges such as toxics or infections in non-lymphoid tissues. Apicobasal polarity is critical to the specialized functions of these epithelia. Indeed, a common feature of epithelial dysfunction is the loss of polarity. Here we review evidence showing that apicobasal polarity regulates the inflammatory response: various polarized epithelia asymmetrically secrete chemotactic mediators and polarize adhesion receptors that dictate the route of leukocyte migration within the parenchyma. We also discuss recent findings showing that the loss of apicobasal polarity increases leukocyte adhesion to epithelial cells and the consequences that this could have for the inflammatory response towards damaged, infected or transformed epithelial cells.

## 1. Introduction

**Figure 1:**
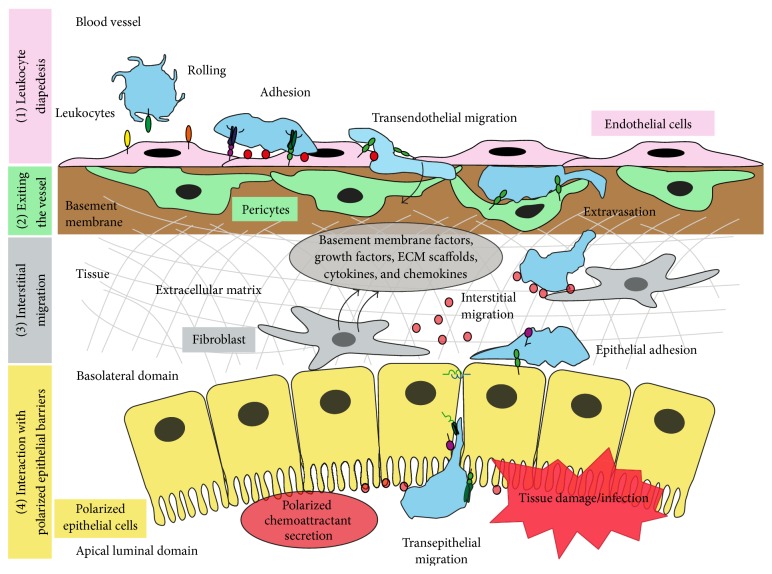
Endothelial and epithelial barriers determine the different stages of leukocyte migration in its journey towards the inflammatory focus in complex tissues. The parenchymal three-dimensional organization contributes to establish an haptotactic and chemotactic gradient (1) Leukocyte adhesion and transendothelial migration or diapedesis. (2) Exiting the vessel. Once they reach the subendothelial space, leukocytes traverse the basement membrane and interact with pericytes, which promote the complete extravasation of immune cells via adhesion receptors. (3) Interstitial migration. Leukocytes switch from a two-dimensional to a less adhesive three-dimensional migration to circumvent topological constraints in the stromal barrier. Fibroblasts help leukocyte navigation by maintaining protein scaffolds and secreting mediators such as cytokines and growth factors, which act as chemotactic cues. (4) Interaction with polarized epithelial barriers. Following haptotactic and chemotactic gradients, leukocytes encounter polarized epithelial cells and often undergo transepithelial migration. The polarized distribution of the adhesive and chemotactic machineries mediates leukocyte guidance through the parenchymal epithelia for immunosurveillance or the clearance of pathogens and dysfunctional cells.

Leukocyte recruitment into the inflamed parenchyma requires successive interactions with cellular and stromal barriers that establish mechanical, chemotactic and haptotactic gradients to guide immune cells towards the inflammatory focus. The first stage of this immune steeplechase, the leukocyte transendothelial migration, is a multi-step cascade of interactions that have been extensively studied in recent years in different vascular beds and experimental models, and some comprehensive reviews on this topic can be found in this special issue [1–5]. The events that follow leukocyte extravasation are perhaps less well characterized, although significant advances have been made with the advent of high-resolution intravital microscopy and the development of more sophisticated culture systems to investigate leukocyte migration and interactions in three dimensions. Particular attention has been paid to elucidating how leukocytes can migrate through the stroma, the way these cells remodel their morphology and sense cues that guide them towards dysfunctional tissue areas. These areas are often made up of polarized parenchymal epithelial cells that form barriers to compartmentalize functions in cavities of the liver, intestine or lungs (Figure 1). Compared to the endothelium, the molecular mechanisms involved in the interaction of infiltrated or tissue-resident immune cells with parenchymal barriers have not been so extensively studied. Polarized epithelial barriers establish two types of interactions. On the one hand, similar to endothelial cells, parenchymal epithelia must guide leukocytes to traverse them in order to reach a localized inflammatory focus, for example, in the lung or intestinal mucosa. These interactions are thus transient and often occur in two directions, from the parenchyma to the lumen and viceversa [6]. On the other hand, these barriers contain damaged or infected cells that are part of the inflammatory focus and the endpoint of the leukocyte migratory journey, so some sort of footprint, which is not completely understood, must exist in these cells to promote a preferential adhesion with infiltrated leukocytes. So far, most of the* in vivo* and* in vitro* approaches to study leukocyte migration across the tissue parenchyma have addressed the role of each single tissue barrier that immune cells encounter in their journey to the inflammatory focus. We believe that successfully combining our current knowledge about leukocyte extravasation, three-dimensional migration through the stroma and the sequential interactions with parenchymal cell barriers, which include adopting unified experimental models, will help shed light on the entire migratory route of each immune cell type and on the specificity of the innate inflammatory responses in each type of tissue.

## 2. The Long Journey towards the Parenchymal Inflammatory Focus after Leukocyte Transendothelial Migration: Leaving the Vessel

Most of the leukocyte efflux from the bloodstream occurs in postcapillary venules, small vessels covered by pericytes and other mural cells and the basement membrane, which are a secondary barrier that extravasating leukocytes have to traverse [9]. Endothelial cells initiate leukocyte extravasation, but subendothelial, leukocyte-pericyte interactions are required for the final egression of leukocytes into the interstitium (Figure 1). Similar to endothelial cells, pericytes express adhesion receptors in response to inflammatory cytokines and establish adhesive tracks at least in the case of neutrophils [10]. Few studies have addressed the contribution of abluminal endothelial surfaces, the basement membrane and the pericyte barriers to leukocyte trafficking into the tissue, but the most recent reports suggest a pro-active role for pericytes in controlling leukocyte navigation into the parenchyma through the intercellular adhesion molecule-1 (ICAM-1), the counter-receptor of *β*2-integrins, which is expressed in pericytes at levels comparable to those in inflamed endothelial cells [10–12]. This interaction occurs mainly in postcapillary venules, where myeloid leukocytes egress through regions between pericytes that have low density of matrix proteins [13]. A subset of these extravasated leukocytes interacts with pericytes surrounding capillaries and arterioles. Pericytes in these microvessels express macrophage inhibitory factor (MIF), which attracts fully extravasated neutrophils and macrophages and, as a consequence, these leukocytes are guided to sites of (sterile) inflammation [12]. Pericytes are not the only perivascular cell type that regulates leukocyte trafficking. Two other cell types have been shown to be critical for neutrophil extravasation: first, resident perivascular macrophages from dermal venules are the main source of neutrophil chemoattractants and secrete the chemokines CXCL1, CXCL2, CCL2, CCL3, and CCL4 in an experimental model of bacterial infection of the skin [14]; second, vessel-associated mast cells secrete the chemoattractants CXCL1 and CXCL2 to induce neutrophil extravasation in a model of intraperitoneal lipopolysaccharide (LPS) stimulation [4].

Leukocyte extravasation constitutes a transition between a two-dimensional migration mode, in the presence of shear stress, to a three-dimensional migration mode upon infiltration into the connective tissue (Figure 1). Immune cells in the interstitium interact with the extracellular matrix and the stromal cells adopting a migratory mode called ameboid, in which cells with a rounded morphology can easily change shape and squeeze between matrix fibers with little stromal remodeling [15]. This ameboid movement requires less adhesiveness than two-dimensional motility, and is mainly based on the ability of the leukocytes to reshape their actomyosin cytoskeleton, emitting pseudopods and inducing contractility at the rear of the cell. Within the heterogeneity of the tissue, leukocytes usually combine adhesion-independent and -dependent motility modes and rapidly adapt their migratory requirements to the microenvironment of the parenchyma [16].

Various fibroblast subtypes reside in the stroma and are adjacent to parenchymal epithelial barriers. Pericryptal fibroblasts, hepatic Ito cells and glomerular mesangial cells support epithelial cell function [17, 18]. These fibroblasts regulate tissue homeostasis and repair by secreting basement membrane factors that contribute to the architecture of the internal epithelial barriers and regulate epithelial cell proliferation and differentiation [19, 20]. Stromal fibroblasts have been attributed a role of maintaining a healthy environment in the lymphoid and non-lymphoid tissue by having an immunomodulatory effect on neighboring endothelial and immune cells [17, 20, 21]. Stromal fibroblasts mediate leukocyte parenchymal navigation through damaged areas by secreting extracellular matrix components as well as cytokines, chemokines and growth factors as soluble chemotactic cues. Direct fibroblast-leukocyte interactions have been investigated in the context of allergic, inflammatory and cancer pathologies [22, 23]. Fibroblasts from patients with rheumatoid arthritis can present autoantigens to infiltrated T-cells and, reciprocally, these cells can induce a proinflammatory status in the fibroblast-like synoviocytes [24, 25]. A probably aberrant leukocyte-fibroblast interaction also occurs in systemic sclerosis, a disease of unknown etiology, in which immune cells cause fibrosis by inducing collagen synthesis in skin fibroblasts [26]. However, the main role of tissue fibroblasts in leukocyte migration seems to be the maintenance of the stromal protein scaffolds and the secretion of mediators that attract or activate migrating immune cells. In conclusion, leukocytes not only follow chemotactic and haptotactic cues within the vessel. The parenchyma establishes a full program of immune cell guidance towards the inflammatory focus. Many cell types orchestrate this program either directly, by interacting with leukocytes, or indirectly, by secreting mediators and extracellular matrix components.

## 3. Reaching the Polarized Epithelia in the Parenchyma

In several tissues, parenchymal cells need to establish lumens to perform specialized functions, including filtration, absorption, secretion and protection. Polarized epithelia and the underlying basement membranes form different mucosal, blood-brain, bile duct or renal barriers, which are exposed to internal cavities and are therefore prone to infections and intense mechanical, toxic and inflammatory stresses. The compartmentalization properties of epithelial barriers arise from the ability to polarize and form intercellular junctions that separate apical from basolateral membrane domains. Columnar epithelial cells place their apical domains facing the lumens that form relatively large tube-shaped cavities [27]. Other polarized epithelia, such as hepatocytes, form smaller apical lumens between adjacent cells, giving rise to smaller tubules that form the bile canaliculus, an intricate network of channels that drains bile into the bile ducts and eventually into the intestine [28]. Importantly, as we shall see below, the compartmentalization properties of the parenchymal epithelia are also fundamental for controlling immune cell trafficking.

Resident leukocytes traverse parenchymal epithelial barriers to survey luminal surfaces exposed to extra-tissular material [29, 30]. Epithelial cell dysfunction can also produce an inflammatory response and additional leukocyte infiltration. Such infiltration may have the final aim of reaching internal cavities, such as the intestinal lumen, to fight luminal pathogens, but leukocytes may also interact with dysfunctional epithelia that themselves constitute the inflammatory focus. Hence, immune cells preferentially contact with impaired epithelial cells to eliminate or receive information from them, so mechanisms must exist to help leukocytes discriminate in the same inflammatory microenvironment between sick cells and inflamed, but still-operative, adjacent cells. On the other hand, leukocytes, particularly neutrophils in the intestine, play an important role in the resolution of epithelial inflammation. Neutrophils transmigrate across inflamed epithelial monolayers and release factors that contribute to tissue repair. It is therefore critical to understand the signals that mediate the leukocyte-epithelium interaction in diverse physiological and pathological scenarios (Figure 1).

## 4. Polarized Secretion of Signals Involved in Leukocyte Attraction to Epithelia

The remarkable compartmentalization occurring between apical and basolateral environments in polarized epithelia led researchers to hypothesize that leukocyte chemoattractants are released in a polarized way. IL-8 is one of the main chemokines driving leukocyte infiltration into the intestine. In an* in vitro* model of human intestinal epithelial cells, Sonnier and colleagues, elegantly showed the different effects on IL-8 secretion caused by polarized TNF*α* stimulation. Whereas basolateral TNF*α* stimulation resulted in secretion of IL-8 from both apical and basolateral membrane regions, apical stimulation induced the secretion of IL-8 exclusively from these surface domains. By combining blocking antibodies, the authors proposed that the receptor 2 for TNF*α* (TNFR2) is apically confined and responsible for polarized IL-8 secretion [31]. Interestingly, the IL-8 receptor CXCR1 is also apically distributed in these cells, suggesting the existence of an autocrine pathway for this chemokine on the luminal side of the intestinal epithelium [32]. Other polarized epithelia, such as endometrial epithelial cells, also vectorially secrete IL-8 depending on the membrane domain receiving the inflammatory stimulus [33]. These cells also release other inflammatory mediators from the apical surfaces such as IL-6 or prostaglandins [33, 34].

The polarized secretion of inflammatory mediators is probably defined by the cell type and the nature and location of the stimulus that induces secretion; indeed, the secretion of IL-8 and other CXC chemokines has been found to be preferentially basolateral in other polarized epithelial beds and with other pathogen-derived stimuli [35–38]. Chemokine secretion polarity has been investigated in more detail with IL-8, but other chemokines involved in lymphocyte attraction to intestinal epithelia, such as interferon *γ*–inducible protein (IP)-10, monokine induced by interferon *γ* (MIG) and MDC/CCL22, are basolaterally secreted and have a differential effect in the basolateral and apical milieu, attracting lymphocytes preferentially from epithelial basolateral membranes, at least for CCL22 [39, 40].

Chemokines are not the only polarized parenchymal chemoattractants. The eicosanoid Hepoxilin A3 (HXA3) is a proinflammatory lipid that is also selectively released from the apical membrane domains of polarized epithelia in intestine and lung. In these two organs, HXA3 is a potent neutrophil chemoattractant that follows the effect of IL-8 to promote neutrophil transepithelial migration [41, 42]. The mechanisms regulating the polarized secretion of this lipid mediator are not well understood, but the apical epithelial marker, multidrug-resistance associated transporter 2 (MRP2) is clearly involved in the apical secretion of HXA3 in the intestine. HXA3 and MRP2 are both induced in experimental models of chronic intestinal inflammation and so constitute a potential therapeutic target [43]. In conclusion, the apicobasal polarity of these chemoattractants is important for efficient leukocyte guidance towards different physiological or pathological inflammatory foci within the complex three-dimensional organization of tissues.

## 5. Apicobasal Polarity of Adhesion Receptors Involved in Leukocyte-Epithelial Cell Interactions

The leukocytes chemoattracted by parenchymal epithelial cells finally make contact. Leukocytes first encounter basolateral epithelial membranes containing fucosylated proteoglycans that can interact with *β*2 integrins [44]. This binding is often the initial step in the process of leukocyte transepithelial migration. It has been reported that macrophages traverse retinal-pigmented epithelial cells from diabetic rats through caveolin-1-positive transcellular pores [45], similar to those found in the transcellular diapedesis of vascular endothelial cells [46, 47]. However, this route of transmigration has not been observed in other polarized epithelial beds. Since transcellular transmigration occurs in the areas of lower membrane resistance [48] the columnar shape that many of the polarized epithelial barriers acquire probably makes it very difficult for immune cells to break through single epithelial cells. Thus, immune cells preferentially negotiate epithelial transmigration following a paracellular route between two cells. The receptor from the immunoglobulin superfamily CD47 is located at lateral cell surfaces and interacts with the signal regulatory protein-(SIRP)-*α* from at least neutrophils, macrophages and dendritic cells [49–51] thereby mediating basolateral-to-apical transepithelial migration. The interaction of these pair of molecules inhibits phagocytosis between immune and cancer cells [51], so these molecules may well regulate the stability of leukocyte-epithelial cell interactions to promote the leukocyte movement across the barrier from the basolateral side. CD47 is also expressed in leukocytes and associates with and regulates their integrin machinery [52]. However, a possible function of CD47 in modulating leukocyte integrin activation from the epithelium has not been reported.

Similar to endothelial cells, some surface receptors forming epithelial cell-cell junctions play a dual role and mediate the process of paracellular transepithelial migration by guiding immune cells across the boundaries between two epithelial cells. Of these, junctional adhesion molecules (JAMs) are paradigmatic receptors from the immunoglobulin superfamily that switch between epithelial cell-cell junctions to heterotypic interactions between endothelial or epithelial cells and leukocytes. The intestinal epithelium mainly expresses JAM-A, JAM-C, JAM4, Coxsackie virus and adenovirus receptor (CAR) and the more distantly related CAR-like membrane protein (CLMP) [53]. It has been reported that the interaction of desmosomal JAM-C with *β*2-integrins mediates PMN transepithelial migration [54]. Desmosomes are more basolaterally located than tight junctions, so this interaction probably precedes that established between epithelial CAR and JAM-L expressed in neutrophils, which also participates in the leukocyte transepithelial migration [53, 55]. In contrast, JAM-A helps maintain the integrity of the epithelial monolayer but does not seem to mediate leukocyte crossing of polarized epithelia [56, 57].

PMNs also proactively contribute to open paracellular spaces during transmigration by secreting serine-proteases such as elastase and cathepsin G. These serine-proteases activate basolateral epithelial protease-activated receptor (PAR)-1 and -2 [58]. PARs are G-protein coupled receptors, which are activated by the proteolytic cleavage of their extracellular domain [59]. Active PARs induce actomyosin-mediated contraction and transiently reduce barrier function to facilitate the transepithelial passage of the immune cell [58].

In summary, the first polarized molecular complexes that leukocytes find during their abluminal interactions with the polarized epithelium are those localized mostly in the lateral cell-to-cell junctions, which play a double function maintaining the epithelial barrier function and facilitating the transmigration of immune cells.

## 6. The Apical Adhesion Machinery

Once leukocytes interact with the epithelial protein machinery exposed in the basolateral domains and traverse the epithelial monolayer, they encounter adhesion proteins confined in the epithelial apical membrane domains. The intercellular adhesion molecule (ICAM)-1 belongs to the immunoglobulin superfamily of transmembrane receptors and interacts with *β*2 integrins from immune cells. Unlike other adhesion receptors that are selectively expressed in endothelial cells, such as E-selectin or VCAM-1, ICAM-1 is broadly expressed in different cell types upon proinflammatory stimulation, including pericytes and parenchymal epithelia. In polarized epithelia, the stimulation of human intestinal epithelial cells with IFN*γ* or by exposure to enteropathogenic bacteria increases apical ICAM-1, suggesting that luminal adhesion of immune cells is important for the inflammatory response to gastrointestinal pathogens [8, 60, 61]. Apical ICAM-1 promotes neutrophil adhesion and crawling on the apical surface of intestinal epithelial cells [61] as well as luminal-to-abluminal neutrophil transepithelial migration [8]. The engagement of apical ICAM-1 signals to the actomyosin cytoskeleton in these cells, inducing contraction and compromising cell barrier function* in vivo* and* in vitro* [61], in a manner comparable to that previously found in endothelial cells [62, 63]. This suggests that a pathological accumulation of PMN cells in the lumen of the intestine may contribute to intestinal barrier dysfunction in response to infections or during chronic proinflammatory diseases. It is of note that ICAM-1-blocking antibodies only have an effect in apical-to-basolateral transepithelial migration and do not affect basolateral-to-apical migration [8]. This suggests that interactions with ICAM-1 probably follow the leukocyte crossing through JAMs and CD47 basolateral surfaces and mediate the return of immune cells from the epithelial lumen towards the parenchyma.

The hyaluronic acid receptor CD44 also belongs to the immunoglobulin superfamily and participates in a wide range of pathological diseases [64]. CD44 displays a notable heterogeneity due to the alternative splicing from the transcription of a single gene, and by the different glycosylation that each isoform undergoes [64–66]. A blocking antibody against the CD44 isoform CD44v6 inhibits the detachment of PMN from the apical membrane domains of polarized intestinal epithelial cells [67, 68]. Although the molecular mechanisms mediating the role of CD44v6 in the apical membrane have not been described in great detail, C44v6 shedding upon PMN interaction seems to be involved. Specific O-glycosylation of this isoform with sialyl Lewis A is required for PMN interaction with CD44v6, but this is not dependent on integrin interactions (Brazil et al., 2013). An increase in expression of the CD44v6 variant in the apical membrane domains has been detected in inflamed colonic mucosa of patients with ulcerative colitis, suggesting a role for CD44 in mobilizing neutrophils to chronic inflammatory lesions in the intestinal tract. In contrast, other epithelial CD44 isoforms, such as CD44v13, which interact with leukocyte *β*2 integrins, are basolaterally expressed in polarized intestinal epithelial cells and so probably mediate other steps in the haptotactic gradient of the intestinal tissue [69].

Two anti-adhesive molecules are also polarized in the apical membrane domains of the parenchymal epithelium. The decay-accelerating factor, CD55, and the ecto-5′-nucleotidase, CD73, are glycosylphosphatidylinositol (GPI) anchored proteins that are apically expressed in intestinal epithelial cells [70, 71]. Similar to CD44v6, CD55 has anti-adhesive properties, although its ligand for infiltrated leukocytes has not been identified yet. CD73 converts AMP to adenosine, which is anti-inflammatory and, in endothelial cells at least, reduces leukocyte adhesion and transmigration [71]. The effect of apical CD73 on leukocyte passage across epithelial barriers has not been investigated in great detail. In epithelial cells, CD73 participates in the generation of adenosine, required for Cl-secretion. Pathological Cl-release causes secretory diarrea, so a role has been proposed for CD73 on PMN-mediated Cl-secretion and diarrea in pathological conditions [70]. CD73 also helps metabolize the ATP secreted by platelets associated with PMN leukocytes that cross the intestinal barriers in some mucosal diseases, which promote bacterial clearance in inflammatory conditions [6, 72]. In conclusion, apical CD73 is central to regulate not only adhesion, but also the effects of immune cells on epithelial barrier function in a pathological context.

Taken together, the data accumulated so far with respect to ICAM-1, CD44v6 and CD55 suggest that they orchestrate the mobilization of immune cells to the luminal side of the intestine, which determines the intensity, duration and resolution of the inflammatory response. However, as mentioned, the specific ligands of CD55 and CD44v6 on luminally-infiltrated leukocytes have not been identified yet and hence, the molecular machinery in leukocytes involved in their detachment from epithelial apical membrane domains remains to be fully elucidated.

## 7. Loss of Apicobasal Polarity and Leukocyte Adhesion to Epithelial Cells

**Figure 2:**
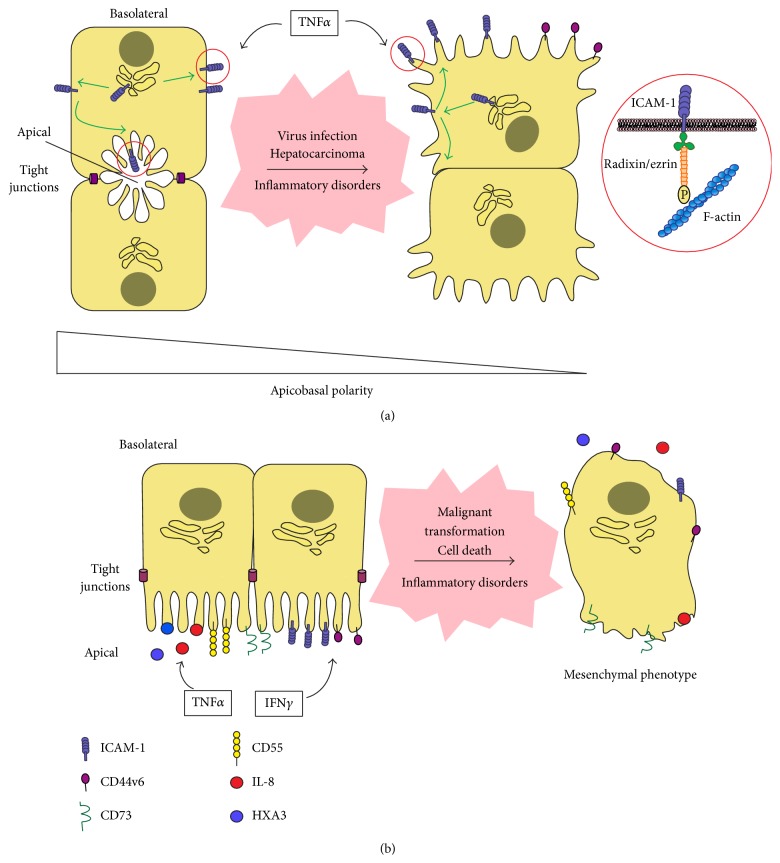
Redistribution of surface receptors and soluble chemoattractants upon loss of apicobasal polarity in epithelial cells. (a) In polarized hepatocytes, ICAM-1 is able to reach the basolateral membrane but is rapidly redirected to the apical membrane domains. Upon hepatic cell depolarization or in response to persistent stimulation with the inflammatory cytokine TNF*α*, ICAM-1 and ezrin-radixin-moesin (ERM) proteins, which connect the receptor to the underlying actin cytoskeleton, are oriented towards the stromal milieu and become accessible to immune cells and small hepatic vessels [7]. Other apically polarized receptors involved in leukocyte adhesion, which also interact with the submembranal actin cytoskeleton, such as CD44 isoforms, should be more exposed to parenchymal immune cells upon loss of apicobasal polarity. (b) Columnar epithelial cells, such as intestinal cells, express on their apical membrane key chemokines and lipid mediators, adhesion receptors and other membrane proteins involved in the mobilization of immune cells to the luminal side of the epithelial barrier during inflammation. The proinflammatory cytokine IFN*γ* is central to increase the expression of some of these receptors, namely ICAM-1 [8]. TNF*α* may also contribute, for example, by stimulating IL-8 secretion from the basolateral or the apical membrane domains. Following epithelial pathologies or cell death, epithelial cells acquire a “mesenchymal phenotype” and therefore, it is plausible to speculate about the loss of polarized distribution of immune cues also in these epithelial cells. However, the relationship between apicobasal polarity and leukocyte adhesion remains to be investigated in columnar epithelial cells.

A common feature of most epithelial pathologies is the loss of apicobasal polarity. Cell death and cancer transformation are the most evident dysfunctions that cause depolarization, but the loss of polarity also underlies inflammatory disorders, such as the inflammatory bowel disease (IBD), which disrupts cell barrier function in the gut mucosal epithelium [73, 74]. In hepatocytes, hepatitis C virus infection disrupts tight junctions and diminishes the canalicular density in infected livers [75]. Some important diseases in kidney, such as polycystic kidney disease directly affect the formation of apical membranes in renal epithelia [76]. Tubular necrosis or autoimmune renal diseases lead to the malfunction of glomerular filtration by altering epithelial tight junctions [77]. No studies have been performed so far on how apicobasal polarity affects the role in the innate immune response of CD44v6, CD55 or CD73. However, CD44v isoforms and CD73 are mesenchymal markers and appear upregulated not only in inflammatory diseases, but also in malignant transformation of epithelial cells such as in hepatocellular carcinoma [78, 79], and in cancer stem cells [80–82]. Thus, changes in the expression and/or the polarized distribution of these plasma membrane proteins could be involved in the interaction of immune cells with epithelial cells undergoing de-differentiation events during cancer or tissue repair (Figure 2).

However, ICAM-1 polarity has received more attention in polarized hepatic cells. Similar to intestinal epithelial layers, ICAM-1 appears apically localized in cholangiocytes and hepatocytes in human tissues from inflammatory diseases that preserve their parenchymal architecture. In contrast, ICAM-1 is depolarized in regions with inflammatory damage and T-cell infiltration, in which parenchymal organization is clearly affected [7]. Interestingly,* in vitro*, upon hepatic cell depolarization, ICAM-1 is dispersed from apical membrane domains, but remains localized at microvilli at the cell surface and becomes accessible to immune cells (Figure 2(a)). Adhesion experiments with human memory T-cells have shown an inverse relation between the apicobasal polarity and the ability to interact with lymphocytes of these hepatic cells. Moreover, loss-of-function experiments by gene silencing or by using blocking antibodies demonstrated that the increase of T-cell adhesion to depolarized hepatic cells is mediated by ICAM-1 exposed upon loss of polarity [7]. Further characterization of the molecular bases of ICAM-1 apical localization indicates that ICAM-1 interaction with the underlying F-actin scaffold is required for the complete confinement of the receptor in the apical plasma membrane domain. In addition, the analysis of the dynamics of photoactivatable ICAM-1-GFP protein has shown that ICAM-1 follows an indirect route of transport toward the apical membrane domain: ICAM-1 can reach the basolateral membrane of polarized hepatic cells, but is rapidly redirected to the apical domains, probably by transcytosis [7]. Thus, polarized, functional hepatic cells have mechanisms, beyond the regulation of protein expression, for depleting ICAM-1 from basolateral membrane domains potentially exposed to hepatic vessels and immune cells. It is of note that, since columnar epithelial cells also confine ICAM-1 in apical membrane domains, loss of apicobasal polarity may have a similar effect on leukocyte-epithelial adhesion, although this remains to be investigated (Figure 2(b)).

The intracellular vesicular trafficking in these polarized cells seems to be important for limiting the accesibility of these receptors to immune cells and thereby their adhesion, at least* in vitro*. Presumably, other apically polarized receptors involved in leukocyte adhesion, which also interact with the submembranal actin cytoskeleton, such as CD44 isoforms, should be more exposed to parenchymal immune cells upon loss of apicobasal polarity caused by cell transformation, damage or infection, and thus, may modulate leukocyte adhesion to those cells that specifically lose their shape within the epithelial barriers. CD44 and ICAM-1 both interact in their cytoplasmic segments with the ezrin-radixin-moesin (ERM) protein subfamily that connects them to filamentous actin [83, 84]. Long-term stimulation with the inflammatory cytokine TNF*α* activates ERMs in different cell types [85, 86]. Interestingly, in polarized hepatic cells, TNF*α* preferentially activates ERMs at the basolateral membrane domains, causing an increase in ICAM-1 exposure, probably by retaining the receptor at the basolateral surface [7] (Figure 2(a)). The molecular requirements for CD44 and ICAM-1 interaction with ERM proteins are quite similar, so it is plausible that inflammatory cytokines may also alter CD44 isoform polarity in different epithelial beds through ERM activation (Figure 2(b)). Therefore, inflammatory cytokines may regulate not only the expression of epithelial adhesion receptors, but also their localization in polarized epithelial cells. Further research on this issue is required, but these findings could have important consequences in the tissue inflammatory response in which infiltrated immune cells need to discriminate operative from dysfunctional, depolarized epithelial cells (Figure 2).

## 8. Concluding Remarks

In this review we have presented evidence of an essential role for epithelial apicobasal polarity in generating a chemotactic and haptotactic gradient for leukocytes in specific tissues containing parenchymal epithelial layers. This evidence comes from studies of the secretion of several chemokines and the polarization of a few receptors involved in the epithelial–leukocyte interaction. Therefore, a major challenge in the near future will be the systematic analysis of the apical and basolateral secretome and “surfaceome” of the main parenchymal cell types. A systems biology approach integrating all this information is likely to reveal the whole set of molecular cues that leukocytes encounter in the inflamed parenchyma during their journey after extravasation. On the other hand, polarized epithelial cells have sophisticated intracellular machinery for sorting surface proteins to each plasma membrane domain. Immunologists have begun to pay closer attention to the endocytic machinery in leukocytes, which appears to be an important player for the immune response. It is time to investigate how vesicular trafficking of receptors and soluble molecules in polarized epithelial cells affect the parenchymal inflammatory response. The effect of inflammatory mediators on this trafficking and, in general, on epithelial apicobasal polarity may provide new therapeutic opportunities for modulating physiological and pathological inflammation in complex tissues.
